# Fates of aflatoxin B_1_ from wheat flour to Iranian traditional cookies: Managing procedures to aflatoxin B_1_ reduction during traditional processing

**DOI:** 10.1002/fsn3.1888

**Published:** 2020-09-19

**Authors:** Razieh Noroozi, Ehsan Sadeghi, Milad Rouhi, Saeede Safajoo, Fatemeh Razmjoo, Giti Paimard, Leila Moradi

**Affiliations:** ^1^ Student Research Committee Department of Food Science and Technology School of Nutrition Sciences and Food Technology Kermanshah University of Medical Science Kermanshah Iran; ^2^ Department of Food Science and Technology School of Nutrition Sciences and Food Technology Research Center for Environmental Determinants of Health (RCEDH) Health Institute Kermanshah University of Medical Sciences Kermanshah Iran

**Keywords:** Aflatoxin B_1_, baking process, fermentation, spices, traditional cookie

## Abstract

Aflatoxin B_1_ (AFB_1_) incidence in cereal, especially in wheat products, is a serious worldwide challenge for human health. The objective of the current study was to survey the effect of various factors, including fermentation times, yeast levels, ingredients, and time/temperature combinations of the baking process on aflatoxin B_1_ (AFB_1_) reduction in order to modify parameters of the traditional cookie‐making process. AFB_1_ levels were analyzed by an HPLC‐fluorescence detector. The results revealed AFB_1_ levels significantly decreased during fermentation (%23.7), depending on an increase in the yeast level (2%) and fermentation time (90 min). Furthermore, there was a significant correlation between pH reduction and AFB_1_ decomposition. However, the formulation of the recipe did not show a significant effect on the detoxification of AFB_1_. The baking temperature increase in an admissible technological range (280°C for 15 min) more effectively reduced AFB_1_ content (%53.9). As a result, the exact control of the traditional process was able to significantly decreased AFB_1_ content as a serious health‐threatening toxin in the final product (%75.9). However, AFB_1_ toxicity reduction should be considered seriously in the raw materials and such products.

## Graphical abstract

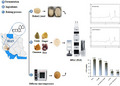

**Abbreviations**
AFB_1_: aflatoxin B1
AFs: aflatoxins
ANOVA: analysis of variance
DON: deoxyvinolavol
FAO: Food and Agriculture Organization
FLD: fluorescence detector
HPLC: high‐performance liquid chromatography
IAC: immunoaffinity columns
MTL: maximum tolerated level
OTA: ochratoxins A
PBS: phosphate‐buffered saline
*S. cerevisiae*: Saccharomyces cerevisiae
SD: standard deviation
WHO: World Health Organization
YGC: yeast extract‐glucose‐chloramphenicol
ZEN: zearalenone

**Highlights**

- Fates of aflatoxin B_1_ from wheat flour to Iranian traditional cookies were investigated.
- Biological (yeast), physical (heat), chemical (spices) methods, on AFB_1_ reduction were surveyed.
- The cookie‐making process was controlled by modifying technological factors on AFB_1_ decrement.
- AFB_1_ levels in all samples were analyzed by reverse‐phase HPLC with a fluorescence detector.

## 1 INTRODUCTION

In recent decades, food contamination with natural or synthetic toxic chemical compounds has become a serious concern worldwide. The health‐threatening risk of mycotoxins has drawn particular attention internationally. The Food and Agriculture Organization (FAO) has reported mycotoxin contamination in agricultural products as one of the most important food safety hazards notably in tropical and subtropical regions (Rahimi, Sadeghi, Bohlouli, & Karami, 2018). The most important classes of mycotoxins are aflatoxins (AFs), ochratoxins A (OTA), deoxyvinolavol (DON), and zearalenone (ZEN) considering their toxicity and economic waste. Among AFs, aflatoxins B_1_ (AFB_1_), B_2_ (AFB_2_), G_1_ (AFG_1_), G_2_ (AFG_2_), M_1_ (AFM_1_), and M_2_ (AFM_2_) are the most prominent and have been observed in a variety of foodstuffs including cereals, nuts, dry fruits, milk, and dairy products (Ismail et al., 2018; Ketney, Santini, & Oancea, 2017). Aflatoxin B_1_ (AFB_1_) is produced as a secondary metabolite of fungal species such as *Aspergillus flavus*, which grow on grains during inappropriate storage (Bol, Araujo, Veras, & Welke, 2016). In addition, wheat flour and wheat flour‐derived products are favored food for fungal growth and mycotoxin production (Generotti et al., 2017).

According to the Annual Agricultural Statistics of Iran, wheat is one of the mostly used cereals in Iran (Sadeghi et al., 2013). Further, wheat‐derived products such as bread, biscuit, cake, and cookies are the main constituents of the human diet not only in Iran but also in many other countries (Bol et al., 2016). Traditional date cookies are one of the most popular and favorite snacks in many provinces of south Iran, especially in Khuzestan Province. Khuzestan is a tropical province with climatic conditions suitable for fungal growth and mycotoxin accumulation. Date cookie is made of wheat flour, traditional date, ginger, cinnamon, and cumin. Owing to the ingredients of the cookie and the climate conditions, a high level of AFB_1_ contamination is probable.

AFB_1_ is a well‐characterized carcinogenic compound which severely contaminates food supplies. The International Agency for Research on Cancer has classified AFB_1_ as the group I health‐threatening carcinogen. Its consumption has also been proven to cause various health problems such as hepatocellular carcinoma (HCC) (Rushing & Selim, 2018). Due to the toxic effects of AFB_1_ on human health, the European Commission has specified a maximum permitted level of 5 µg/kg in foodstuffs and 2 µg/kg in cereals and breakfast cereals (European Commission (EC), 2006). Moreover, the maximum tolerated level (MTL) of AFB_1_ and total AFs for foodstuff in Iran are 5 and 10 µg/kg, respectively (Institute of Standards and Industrial Research of Iran (ISIRI), 2002). Therefore, the World Health Organization (WHO) has a great demand for optimizing the production processes to partially or completely eliminate the toxin to ensure the safety of the final product (Karlovsky et al., 2016). In addition, strategies of AFB_1_ detoxification in foods and feeds not only have a direct impact on human and animal health, but also can increase the safety of dairy products via decreasing AFM_1_, as the main hydroxylated compound derived from AFB_1_ contained in lactating animals feed (Corassin, Bovo, Rosim, & Oliveira, 2013; Gonçalves et al., 2017).

Recent advances in food processing, including physical, chemical, and biological manipulations have been developed to guarantee the final level of AFB_1_ under standard limitations in food products (Cusato et al., 2013). Although aflatoxins are great heat‐stable (Jager, Tedesco, Souto, & Oliveira, 2013), food processing can cause the destruction of varying degrees of aflatoxins in food products. Generally, the efficiency of thermal treatments for mycotoxins detoxification in food is highly related to factors like heating temperature, time of exposure, fermentation, pH, and also concentration and type of toxin (Ismail et al., 2018; Milani & Maleki, 2014).

The AFs occurrence and the effect of the food processing on detoxification have been studied in the previous researches (Ahmadi, 2020; Hajmohammadi et al., 2020; Milani, Nazari, Saman, Bamyar, & Maleki, 2018; Rastegar et al., 2017). However, as far as we know, there are little data about AFB_1_ incidence in wheat flour and its derived products in Iran. Furthermore, there is no study regarding the influence of its ingredients and processing agents on final AFB_1_ content in the traditional Iranian bakery products such as date cookies. Thus, the objectives of this survey were to investigate the effect of biological (fermentation), physical (thermal process), and chemical (spices) treatments on AFB_1_ reduction during the traditional cookie‐making from the wheat flour samples of Khuzestan (southern part of Iran) and to determine an optimal production process to produce the final product with the lowest possible level of AFB_1_.

## 2 MATERIALS AND METHODS

### 2.1 Chemicals and reagents

Crystalline AFB_1_ was bought from Sigma‐Aldrich (USA). For the clean‐up step of samples, AFLA‐test immunoaffinity column (LC Tech GmbH) was used following the manufacturer's advice. Whatman filter paper No.1 was supplied. Methanol, acetonitrile, water, and phosphate‐buffered saline (PBS) were obtained from Merck. All solvents were of HPLC grade. The water used in all experiments was generated by a water purification system (Millipore).

The AFB_1_ stock solution was prepared by adding 1 mg AFB_1_ to 10 ml HPLC grade methanol, which was then kept at −20°C and was brought to room temperature before use. Then, the standard working solutions were prepared by adding adequate dilutions of the stock solution at methanol: water ratio of 40:60 v/v ranging from 0.02 to 20 ng/ml and were kept at 4°C. The working standard was renewed every two weeks. To minimize the health risks caused by AFs pollution, all laboratory glassware used was washed by sodium hypochlorite (5%) before discarding. The Iranian wheat flour samples were randomly bought from the local bakeries and *Saccharomyces cerevisiae* was purchased from Khamir Mayeh‐Khuzestan (Dez mayeh, Iran). Moreover, dates, ginger, cinnamon, cumin, and other additives were obtained from markets in Khuzestan Province, Southwest of Iran.

### 2.2 Spiking process of wheat flour

The initial content of AFB_1_ in wheat flour was evaluated by high‐performance liquid chromatography (HPLC) with a fluorescence detector (FLD). The mean AFB_1_ concentration detected was approximately 3.14 µg/kg in the wheat flour samples, which was probable to decline to a level below the detection limits of the methods during the production process used in this research. Further, this toxin was not found in the analyzed batches of other formulation additives. To simulate the possible highest level of AFB_1_ contamination, a part of blank wheat flour was contaminated by adding an AFB_1_ methanolic solution with a concentration of 1,000 ng/ml (Institute of Standards and Industrial Research of Iran (ISIRI), 2002). No change was observed in the flour texture using this ratio of organic solution/flour compared to the blank samples. The flour spiking process was done in a glass bottle. Then, each part of the spiked flour was homogenized and the mycotoxin solvent was evaporated. The contaminated samples were analyzed before processing, whose levels were compatible with the recovery rate of this method (Bol et al., 2016).

### 2.3 Dough fermentation process

The dough was created with contaminated flour (25 g), water (17 ml), and *Saccharomyces cerevisiae* (commonly known as baker's yeast). To investigate the effects of fermentation on AFB_1_ contamination, the samples were prepared with two levels of baker's yeast (1%, 2% w/w). The yeast survivability was evaluated by microbial examination as CFU per gram of dry matter. The yeast was mixed with water and the yeast cell suspension was counted by the plate counting method on the yeast extract‐glucose‐chloramphenicol (YGC) agar (Merck, Germany). The average number of viable yeast cells was 12 × 10^10^ (CFU/g) in triplicate after 5 days of incubation at 25°C (Rad & Kasaie, 2017). The dough samples (5 and 10 µg/kg) were physically mixed until they shaped an elastic and nonsticky structure. The prepared samples were fermented in the oven at 35°C for 30, 60, and 90 min. After extraction and purification, the remaining levels of AFB_1_ were detected. According to the results, the optimum yeast concentration and fermentation time were determined.

### 2.4 pH determination

To determine the pH value of dough samples containing 10 µg/kg AFB_1_ during the fermentation process, 5 g of the dough samples were mixed with 45 ml water and vortexed (3 min). After that, the pH value was determined by a pH meter from Mettler Toledo (Greisensee, Switzerland). The analysis was performed in triplicate (Peng et al., 2015).

### 2.5 Cookie preparation and baking process

In the second step, the optimized fermentation conditions were applied to all samples and they were prepared in a laboratory according to the Iranian traditional cooking method. In order to prepare date cookies, 25 g of contaminated wheat flour was mixed with the baker's yeast (2%) suspended in water (17 ml), and the following ingredients, including a mixture of spices with equal ratios (2 g) and date paste (5 g), were added. The samples were prepared with 5 and 10 µg/kg AFB_1_ respectively, and the weight of each piece was estimated to be at least 50 g. After preparation of samples, they were subjected to the traditional thermal processing of date cookies using different temperature/time combinations, including baking at 160°C for 45 min, 220°C for 25 min, and 280°C for 15 min, to obtain a well‐baked final product. All samples were milled by a Retsch cutting mill (Hann, Germany) to achieve homogenized powder and were stored at − 20°C under dark conditions until they were used for mycotoxin analysis.

### 2.6 Sample extraction and analysis

After the baking process, sample preparation for AFB_1_ analysis was done according to the manufacturer's immunoaffinity columns (IAC) instructions (LCTech GmbH, Germany). One gram NaCl was added to 20 g of the thoroughly homogenized powder of samples. Then, 100 ml methanol: water (8:2) mixture was added to perform extraction by a laboratory blender at high speed. After 3 min stirring, the sample was filtrated twice, and 14 ml of the filtered sample was diluted with 86 ml PBS solution. The IAC was equilibrated by passing 10 ml PBS solution at a flow rate of 1–2 drops per second. The diluted extract was then passed through that. The column was eluted with 10 ml distilled water. Afterward, AFB_1_ was washed with 2 ml methanol. The gathered solvent was evaporated with nitrogen gas at room temperature, reconstituted by 1 ml methanol: water (1:1, v/v), and saved at − 18°C until analysis. Eventually, 100 μl elution was injected into the HPLC (Imperato, Campone, Piccinelli, Veneziano, & Rastrelli, 2011). The measurement of AFB_1_ levels was carried out in triplicate by an HPLC system Knauer (AZURA) equipped with a fluorescence detector (RF‐20A) with photochemical postcolumn derivatizsation of UV system (LCTech). The wavelengths of the fluorescence detector were set at 329 and 460 nm for excitation and emission, respectively. The HPLC column was Knauer C18 analytical column (250 4.6 mm I.D., 5 mm) with a precolumn, and the temperature of the column was held at 40°C. The mobile phase used was acetonitrile/water (90:10) at a flow rate of 1.5 ml/min.

### 2.7 Validation of method

The HPLC quality control parameters like the calibration curve linearity, the limit of detection (LOD), the limit of quantification (LOQ), and repeatability of AFB_1_‐contaminated samples were obtained to validate the method developed in this study. Moreover, the recovery percentage of AFB_1_ in samples was measured to confirm the accuracy of the method. The linear calibration curve was acquired by analyzing the linear least square regression of the peak area against the concentration of AFB_1_. The calibration plot was linear for AFB_1_ in the concentration range of 0.02–20 μg/kg, which showed a correlation *R* ^2^ of 0.996. The equation calibration curve was obtained by the following equation: y = 39.359x + 14.592. LOD and LOQ were 0.005 μg/kg and 0.015 μg/kg, respectively. Due to the precision of the method applied, flour, dough, and cookie were experimentally spiked with 5 and 10 μg/kg concentrations of AFB_1_. The recovery rate and standard deviation (*SD*) were calculated (*n* = 3) afterward (Table 1). The mean recovery rate of the chromatographic analysis of AFB_1_ for two concentrations indicated the method applied was highly reliable for AFB_1_ detection in these samples.

**Table 1:** Recovery values of AFB_1_ spiked in various stages of cookie‐making at two levels: low (5 μg/kg) and high (10 μg/kg)

Recovery and standard deviation (%)			
Spiked level (μg/kg)	Wheat flour	Dough	Cookie
Low	98.4 ± 0.09	92.4 ± 0.11	97.4 ± 0.09
High	98.6 ± 0.13	92.2 ± 0.12	99.1 ± 0.09

### 2.8 Statistical analysis

All statistical analyses were done by SPSS (version 16.0) software. Results are presented as mean ± standard deviation. Evaluation of the AFB_1_ levels was performed by one‐way analysis of variance (ANOVA) together with Duncan's test. Pearson's correlation coefficient was used to determine the correlation between AFB_1_ level and pH. Independent samples *t* test was used to compare the means between the two groups. *p* < .05 was considered significant for all experimental data.

## 3 RESULTS AND DISCUSSION

### 3.1 Effect of fermentation on AFB_1_

Results of AFB_1_ changes during dough fermentation, as one of the main steps of cookie‐making, are shown in Table 2. AFB_1_ concentrations in the mixed dough were approximately similar to those of the flour. Based on our results (Table 2), the fermented dough samples showed a significant decrement in AFB_1_ levels compared with the control dough sample with an increase in the yeast content from 1% to 2% w/w (*p* < .05). Moreover, the longer the time of fermentation (from 30 to 90 min), the more the reduction of AFB_1_. Furthermore, the results showed that fermentation had a more efficient effect on reducing the higher concentration of AFB_1_ (10 μg/kg) compared to the lower concentration (5 μg/kg). The lowest amount of AFB_1_ (7.63 μg/kg) was found in the sample with 2% yeast and 90 min fermentation when the initial concentration of toxin was high.

**Table 2:** Concentration of AFB_1_ (μg/kg) during dough fermentation with various yeast levels in different times

		Fermentation time (min)					
		30 min		60 min		90 min	
Yeast content (%)	Spiked concentration (μg/kg)	AFB_1_ (μg/kg)	Reduction of AFB_1_ (%)	AFB_1_ (μg/kg)	Reduction of AFB_1_ (%)	AFB_1_ (μg/kg)	Reduction of AFB_1_ (%)
0%	5	4.62 ± 0.026^Aa^	%7.6	4.6 ± 0.026^Aa^	%8.0	4.59 ± 0.026^Aa^	%8.2
	10	9.22 ± 0.026^Aa^	%7.8	9.2 ± 0.026^Aa^	%8.0	9.19 ± 0.01^Aa^	%8.1
1%	5	4.53 ± 0.03^Aa^	%9.4	4.44 ± 0.043^Bb^	%11.2	4.35 ± 0.036^Bb^	%13.0
	10	8.94 ± 0.036^Ba^	%10.6	8.75 ± 0.036^Bb^	%12.5	8.54 ± 0.036^Bc^	%14.6
2%	5	4.26 ± 0.045^Ba^	%14.8	4.12 ± 0.026^Cb^	%17.6	3.98 ± 0.036^Cc^	%20.4
	10	8.29 ± 0.026^Ca^	%17.1	7.95 ± 0.052^Cb^	%20.5	7.63 ± 0.036^Cc^	%23.7

Note

Means ± standard deviations of triplicate independent experiments are shown. Different superscript capital letters within a column indicate statistically significant differences in AFB_1_ changes at different yeast levels (in the same concentration). Different superscript small letters within a row indicate statistically significant differences in AFB_1_ changes at different times; if they have a common letter, they are not significantly different.

The results of this survey showed that the amount of yeast and the fermentation time were effective parameters for the AFB_1_ reduction during fermentation. Therefore, these results are in accordance with the results reported by El‐Banna and Scott (1983). They reported that, on average, 19% of the added AFB_1_ was destroyed after the fermentation process. A similar order was reported by Uma Reddy, Gulla, and Nagalakshmi (2010). They found that a significant fall in AFB_1_ content in a higher fermentation time. These results are in agreement with those obtained in this study. In another study, researchers reported that the fermentation process under 30°C for 50 min using 1% yeast reduced the AFB_1_ level by 6% (Bol et al., 2016), which is not as effective as that of the present study. It could be due to the lower yeast concentration and shorter fermentation time. Moreover, other studies (Mozaffary, Milani, & Heshmati, 2019; Valle‐Algarra et al., 2009) on mycotoxin reduction during wheat bread fermentation showed increased in fermentation time and yeast concentration could have more effect on reducing mycotoxins, that was in good agreement with our results.

Scientists believe that the biological methods can not only decrease the toxic component but also maintain the nutritional values of foods and therefore are considered a safe way for aflatoxin detoxification in the food industry (Gonçalves et al., 2020; Peng et al., 2018). *S*. *cerevisiae* is one of the most effective microorganisms to remove or degrade AFB_1_ in the food products. Furthermore, it has shown the potential ability to reduce AFM_1_ in the dairy industry (Gonçalves et al., 2017, 2020).

S. *cerevisiae* has capable of reducing mycotoxins under soft conditions without using any chemical substances, by binding mycotoxins to its cell wall (Karazhyan, 2017). Many researchers (Aazami, Nasri, Mojtahedi, & Battacone, 2019) have previously reported that *S. cerevisiae* is able to change the chemical bonds between AFs and food proteins by binding toxins to its own cell wall polysaccharides (such as mannose and glucan) and to transform AFs into less toxic substances by releasing degrading enzymes, lactic acid, ethanol, and CO_2_. This supposition was validated by Shetty, Hald, and Jespersen (2007). They observed that even inactivated *S. cerevisiae* cells could effectively reduce the amount of AFB_1_ via treatment at 120°C. These results suggest that the heat‐resistant enzymes of *S. cerevisiae* are probably important in the destruction of aflatoxin. In another study, the effect of fermentation process on detoxification in food products was investigated and results indicated that AFB_1_ could be absorbed by the baker's yeast cell wall (Joannis‐Cassan, Tozlovanu, Hadjeba‐Medjdoub, Ballet, & Pfohl‐Leszkowicz, 2011). Based on the published results, the yeast cell wall is able to absorb AFB_1_ up to 29%. The results of the present study indicated more decrement in the amount of toxin in samples with a higher initial concentration of AFB_1_.

Researchers have reported since the binding of AF to mannoprotein (mannan), one of the main cell wall ingredients of S. cerevisiae, is physical (hydrogen, and van der Waals), it increases over time and at higher toxin concentrations (Shetty et al., 2007). Increasing the initial concentration of the toxin actually increases the substrate for the reaction between AF and mannan. Consequently, these bindings are made with greater intensity and speed, leading to an increase in the percentage of toxin reduction. These results are in line with those of Karazhyan et al., indicating the amount of adsorbed toxin increased with a rise in the concentration of toxins, but this amount of adsorption was not significant at different concentrations of the toxin (Karazhyan, 2017).

Moreover, different chemical and physical features of the toxins, including their charge distribution, polarity, solubility, size, and shape dissociation, affect the adsorption process during fermentation; however, its mechanism is still unknown (Rastegar et al., 2017; Uma Reddy et al., 2010).

In general, the differing amounts of AFB_1_ in dough preparation stages, including kneading and fermentation, are probably explained by the variation of initial yeast content, the time and temperature of fermentation, and the effect of enzymes like a‐amylase, glucose‐oxidase, xylanase, cellulase, and protease (Vidal, Sanchis, Ramos, & Marin, 2016).

### 3.2 Association of pH with AFB_1_ decrement

**Figure 1:**
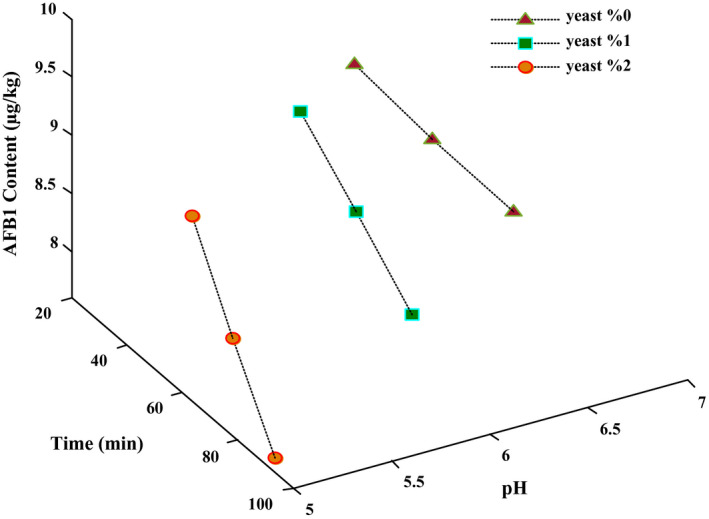
The correlation between concentration changes of AFB1 and pH during dough fermentation

According to Figure 1, as the fermentation time and yeast level increase, the pH and toxin content of dough samples decrease; therefore, the highest reduction of AFB_1_ (7.63 μg/kg) was detected in the fermented dough sample with the lowest pH. In addition, there was a significant correlation between pH reduction and AFB_1_ decomposition (Pearson's coefficient = 0.998, *p* < .05). The reduction of AFB_1_ levels could be because of the metabolites created during the fermentation procedure or the binding ability of this toxin to yeast wall. The pH drop of dough during fermentation, due to the production of organic acids (succinic acid and carbon dioxide) by the yeasts, resulted in the decomposition or inactivating of AFs (Milani & Heidari, 2017). The pH reduction and acidic condition by hydration of AFB_1_ at the terminal furan ring could decompose this toxin to its hemiacetal form (AFB2a), whose toxicity is 1/200 less than AFB_1_. As demonstrated by previous investigations, AF treatment with strong and diluted acids leads to the conversion of AFB_1_ to less toxic products (Adebo, Njobeh, Gbashi, Nwinyi, & Mavumengwana, 2017; Aiko, Edamana, & Mehta, 2016). Based on the results of these studies, the organic acids and other compounds produced by the yeast can influence AFB_1_, which is an effective way for detoxification of AFs in comparison with other chemical methods (Milani & Heidari, 2017).

### 3.3 The effect of formulation on AFB_1_ changes

**Figure 2:**
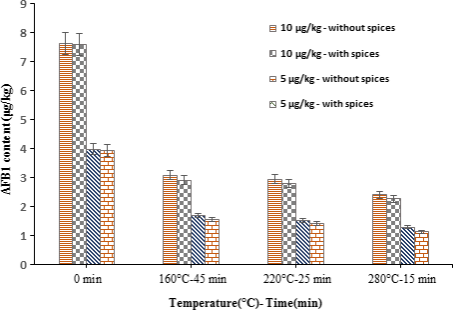
The effect of formulation on the concentration of AFB1 (μg/kg) during cookie baking process. Standard deviation in mycotoxin levels is presented by error bars. Statistical significance was determined by ANOVA and *t* test (*p* < .05)

Traditionally, some spices such as ginger, cinnamon, and cumin are commonly used as condiments to add flavor to food products. These aromatic herbs contain a lot of phenolic compounds, flavonoids, alkaloids, steroids, terpenoids, saponins, and tannin, agents that offer antioxidant and antibacterial properties to them and make them capable of removing food toxins and biological contaminations (Lv et al., 2012; Noroozi et al., 2019). The effect of spices on the AFB_1_ content of cookies after thermal processing is exhibited in Figure 2.

Various studies have approved the detoxification ability of herbal compounds, spices, and other ingredients used in food production, some of which are mentioned below. A study indicated the essential oils of orange and lemon lead to 90% reduction in AF production (Hasan, 2000). In agreement with these results, other researchers reported that ajwain extract (carom), used as a spice was able to destroy AFs, by modifications of the lactone ring in the aflatoxin structure (Velazhahan et al., 2010). Several studies (Panda & Mehta, 2013; Vijayanandraj et al., 2014) have shown that extracts of medicinal plants such as Ocimum *tenuiflorum* and aqueous extracts of vasaka leaves (*Adhatoda vasica*) detoxify AF even at room temperature.

Spices are used as a natural way to reduce food contamination since these compounds are environmentally friendly and they can be safely used against microbial, fungal, and toxic contaminations (Ismail et al., 2018; Velazhahan et al., 2010). Our results showed that the spices, such as cumin, ginger, and cinnamon, used in the formulation of cookies did not significantly reduce AFB_1_ content (*p* > .05). This result could be attributed to the use of plant powder, which was less effective than its essential oil and extract. This issue, in particular, requires further research.

### 3.4 The effect of baking process on AFB_1_ levels

To investigate the effect of different baking protocols as the final step of cookie‐making, the optimized conditions for dough fermentation (2% yeast with 90 min fermentation) were applied and the samples containing 7.63 and 3.98 μg/kg AFB_1_ were then subjected to various baking conditions (temperature/time combination) in the oven. Next, the samples with or without fermentation were compared. According to the data (Table 3), all three combinations of the baking process could efficiently decrease the initial contamination level, but the maximum reduction in AFB_1_ concentration was expected to occur at 280°C because its baking temperatures are higher than those of others.

**Table 3:** The effect of fermentation and baking process (three main protocols) on the changes of AFB_1_ concentration

Baking stage	Spiked concentration (μg/kg)	Treatment A^a^		Treatment B^b^	
		Concentration of AFB_1_ (μg/kg)	Reduction of AFB_1_ (%)	Concentration of AFB_1_ (μg/kg)	Reduction of AFB_1_ (%)
D (0 min)	5	4.62 ± 0.026^A^	7.6%	3.98 ± 0.036^A^	%20.4
	10	9.22 ± 0.026^A^	7.8%	7.63 ± 0.036^A^	%23.7
C (160ᵒC−45 min)	5	2.60 ± 0.036^B^	48%	1.69 ± 0.026^B^	%66.2
	10	5.30 ± 0.036^B^	47%	3.08 ± 0.036^B^	%69.2
C (220ᵒC–25 min)	5	2.45 ± 0.04^C^	51%	1.52 ± 0.03^C^	%69.6
	10	5.11 ± 0.026^C^	48.9%	2.94 ± 0.043^C^	%70.6
C (280ᵒC–15 min)	5	2.13 ± 0.036^D^	57.4%	1.29 ± 0.02^D^	%74.2
	10	4.61 ± 0.034^D^	53.9%	2.41 ± 0.045^D^	%75.9

Note

The treatments were nominated by D (Dough before baking process) or C (cookie after the baking process), ^a^treatment (A): samples without yeast, ^b^treatment (B): samples with 2% yeast and optimal fermentation conditions^.^ Means ± standard deviations of triplicate independent experiments are shown. Different superscript capital letters in the same concentration within a column indicate statistically significant differences (*p* < .05) among values; if they have a common letter, they are not significantly different.

The baking process is an important physical procedure for the destruction of AFB_1_, a toxin with low molecular weight and small molecular size that mainly opens the lactone rings and makes AFs sensitive to other reactions altering the binding properties of terminal furan ring. Such structural changes could ultimately accelerate the degradation reactions of AFs. Moreover, heat treatment increases the toxin adsorption ability of these cells by creating active adsorption sites in the cell wall of saccharomyces (Milani et al., 2018).

Different experimental researches (Bullerman & Bianchini, 2007; Generotti et al., 2017; Rahimi et al., 2018) have revealed that an optimal combination of time/temperature processing, especially at higher baking temperatures, can considerably reduce the AFB_1_ contamination. Based on our results, the major effect of the baking time/temperature was reported for the fermented samples with higher initial AFB_1_ levels whose baking process was done at 280°C for 15 min. Thermal destruction products and mycotoxin‐conjugated forms were not detected by the analytical procedure applied in the present study.

Based on the results obtained, the highest reduction (%) of AFB_1_ in samples with 5 and 10 μg/kg AFB_1_ after fermentation were 74.2% and 75.9% and the highest reduction (%) in samples without yeast were 57.4% and 53.9% respectively. Detailed results are presented in Table 2. The amount of AFB_1_ reduction was greater than those reported in most studies. Thus, the higher reduction in our study was due to the use of more effective parameters during product preparation and processing (samples with 2% yeast, 90 min fermentation time, and baking process at 280°C for 15 min). Moreover, due to the size and shape of the cookies, the heat penetration may be higher. Other data obtained from studies on bread, biscuit, and cake regarding the impact of baking time/temperature on the removal of mycotoxins were in accordance with our results (Generotti et al., 2017; Valle‐Algarra et al., 2009).

A result similar to our study was reported by Banna et al. They found that 55% of the AFB_1_ content in flour was reduced in the Egyptian bread after fermentation and the subsequent baking (El‐Banna & Scott, 1983). Furthermore, Bol et al., (2016) investigated the processing effect on AFB_1_ levels in bakery products and they reported 36, 40, and 70% mycotoxin reduction in bread, biscuit, and cake during processing, respectively. Similarly, Hwang et al. surveyed the effects of different cooking procedures on AF toxicity reduction and showed 50% and 90% decrement in AFB_1_ content in wheat due to heat treatment at 150 and 200°C, respectively. Further, the traditional processing of the steamed bread and Sujebi (wheat flakes soup) decreased the AFB_1_ content by 43% and 71%, respectively (Hwang & Lee, 2006). Moreover, the results of a study by Tawila et al. showed that the mean AFB_1_ concentration was 31.98% lower in bread than in flour (El‐Tawila, Ibrahim, Gomaa, & Omar, 2003). In addition, some studies (Generotti et al., 2017; Rahimi et al., 2018; Valle‐Algarra et al., 2009) have reported similar results for the reduction of other mycotoxins during the baking process.

Pursuant to a literature review by Schaarschmidt et al., AFB_1_ decrease during the baking procedure is highly dependent on various factors, such as the initial AFB_1_ content in flour, dough combination containing microorganisms and enzymes, fermentation rate, and baking temperature (Schaarschmidt & Fauhl‐Hassek, 2018). Based on the results of this study, the baking process was not capable of entirely eliminating AFB_1_ from the investigated samples. However, the AFB_1_ level was significantly lower in the baked cookies than in dough and flour (*p* < .05).

## 4 CONCLUSION

The current study investigated the effect of the modifications of technological factors and other ingredients in the recipe of traditional date cookie on the final AFB_1_ content. Based on the data obtained, decrement of pH value after fermentation under different conditions decreased AFB_1_ during cookie manufacturing, especially when the initial content of toxin was high in the samples. The results indicated that the baker's yeast content, time, and pH values involved in fermentation play a basic role in AFB_1_ reduction. The formulation of the recipe, despite its phenolic content, and antioxidant properties did not show a significant effect on AFB_1_ decrement. Enhancement of baking temperature/time at an admissible technological range especially in products fermented prior to the baking process effectively reduced AFB_1_ content. In general, it was found that AFB_1_ decreased during different treatments (various fermentation times, yeast levels, and time/temperature combinations of the baking process) applied in this study as a detoxification process. The present study provides an optimized preparation protocol for traditional cookies and demonstrates how exact control of the traditional process may decrease AFB_1_ impact as a serious health‐threatening toxin in the final product. Such studies are crucial to adopt suitable management strategies to ensure the safety of the local food supply in the country.

## CONFLICT OF INTEREST

The authors declare that there is no conflict of interest.

## ETHICAL APPROVAL

This article does not involve any human or animal experiments.

## References

[fsn31888-bib-0001] Aazami, M. H. , Nasri, M. H. F. , Mojtahedi, M. , & Battacone, G. (2019). Effect of yeast cell wall and (1→ 3)‐β‐D‐glucan on transfer of aflatoxin from feed to milk in Saanen dairy goats. Animal Feed Science and Technology, 254, 114191 10.1016/j.anifeedsci.2019.05.014

[fsn31888-bib-0002] Adebo, O. , Njobeh, P. , Gbashi, S. , Nwinyi, O. , & Mavumengwana, V. (2017). Review on microbial degradation of aflatoxins. Critical Reviews in Food Science and Nutrition, 57(15), 3208–3217. 10.1080/10408398.2015.1106440 26517507

[fsn31888-bib-0003] Ahmadi, E. (2020). Potential public health risk due to consumption of contaminated bovine milk with aflatoxin M1 and Coxiella burnetii in the West of Iran. International Journal of Dairy Technology, 57(15), 3208–3217. 10.1080/10408398.2015.1106440

[fsn31888-bib-0004] Aiko, V. , Edamana, P. , & Mehta, A. (2016). Decomposition and detoxification of aflatoxin B1 by lactic acid. Journal of the Science of Food and Agriculture, 96(6), 1959–1966. 26095453 10.1002/jsfa.7304

[fsn31888-bib-0005] Bol, E. K. , Araujo, L. , Veras, F. F. , & Welke, J. E. (2016). Estimated exposure to zearalenone, ochratoxin A and aflatoxin B1 through the consume of bakery products and pasta considering effects of food processing. Food and Chemical Toxicology, 89, 85–91. 10.1016/j.fct.2016.01.013 26807886

[fsn31888-bib-0006] Bullerman, L. B. , & Bianchini, A. (2007). Stability of mycotoxins during food processing. International Journal of Food Microbiology, 119(1–2), 140–146. 10.1016/j.ijfoodmicro.2007.07.035 17804104

[fsn31888-bib-0008] Corassin, C. , Bovo, F. , Rosim, R. , & Oliveira, C. (2013). Efficiency of Saccharomyces cerevisiae and lactic acid bacteria strains to bind aflatoxin M1 in UHT skim milk. Food Control, 31(1), 80–83. 10.1016/j.foodcont.2012.09.033

[fsn31888-bib-0009] Cusato, S. , Gameiro, A. H. , Corassin, C. H. , SantAna, A. S. , Cruz, A. G. , Faria, J. A. F. , & de Oliveira, C. A. F. (2013). Food safety systems in a small dairy factory: Implementation, major challenges, and assessment of systems' performances. Foodborne Pathogens and Disease, 10(1), 6–12. 10.1089/fpd.2012.1286 23153286

[fsn31888-bib-0010] El‐Banna, A. , & Scott, P. (1983). Fate of mycotoxins during processing of foodstuffs I‐aflatoxin B1 during making of Egyptian bread. Journal of Food Protection, 46(4), 301–304. 10.4315/0362-028X-46.4.301 30913594

[fsn31888-bib-0011] El‐Tawila, M. , Ibrahim, N. , Gomaa, N. , & Omar, R. (2003). The effect of bread making steps on the degradation of aflatoxins produced as a result of single inoculation with *Aspergillus flavus* and combined inoculation with *Aspergillus flavus* and *Aspergillus ochraceus* . The Journal of the Egyptian Public Health Association, 78(5–6), 373–386. 17219901

[fsn31888-bib-0007] European Commission (EC) (2006). Setting of maximum levels for certain contaminants in foodstuffs. *regulation*(1881), 5‐24.

[fsn31888-bib-0012] Generotti, S. , Cirlini, M. , Šarkanj, B. , Sulyok, M. , Berthiller, F. , Dall'Asta, C. , & Suman, M. (2017). Formulation and processing factors affecting trichothecene mycotoxins within industrial biscuit‐making. Food Chemistry, 229, 597–603. 10.1016/j.foodchem.2017.02.115 28372220

[fsn31888-bib-0013] Gonçalves, B. , Gonçalves, J. , Rosim, R. , Cappato, L. , Cruz, A. , Oliveira, C. , & Corassin, C. (2017). Effects of different sources of Saccharomyces cerevisiae biomass on milk production, composition, and aflatoxin M1 excretion in milk from dairy cows fed aflatoxin B1. Journal of Dairy Science, 100(7), 5701–5708. 10.3168/jds.2016-12215 28478008

[fsn31888-bib-0014] Gonçalves, B. L. , Muaz, K. , Coppa, C. F. S. C. , Rosim, R. E. , Kamimura, E. S. , Oliveira, C. A. F. , & Corassin, C. H. (2020). Aflatoxin M1 absorption by non‐viable cells of lactic acid bacteria and *Saccharomyces cerevisiae* strains in frescal cheese. Food Research International, 136, 109604– 10.1016/j.foodres.2020.109604 32846626

[fsn31888-bib-0015] Hajmohammadi, M. , Valizadeh, R. , Naserian, A. , Nourozi, M. E. , Rocha, R. S. , & Oliveira, C. A. (2020). Composition and occurrence of aflatoxin M1 in cow's milk samples from Razavi Khorasan Province. Iran. International Journal of Dairy Technology, 73(1), 40–45.

[fsn31888-bib-0016] Hasan, H. (2000). Patulin and aflatoxin in brown rot lesion of apple fruits and their regulation. World Journal of Microbiology and Biotechnology, 16(7), 607–612.

[fsn31888-bib-0017] Hwang, J.‐H. , & Lee, K.‐G. (2006). Reduction of aflatoxin B1 contamination in wheat by various cooking treatments. Food Chemistry, 98(1), 71–75. 10.1016/j.foodchem.2005.04.038

[fsn31888-bib-0018] Imperato, R. , Campone, L. , Piccinelli, A. L. , Veneziano, A. , & Rastrelli, L. (2011). Survey of aflatoxins and ochratoxin a contamination in food products imported in Italy. Food Control, 22(12), 1905–1910. 10.1016/j.foodcont.2011.05.002

[fsn31888-bib-0041] Institute of Standards and Industrial Research of Iran (ISIRI) . (2002 & Maximum tolerated limits of mycotoxins in foods and feeds. *National Standard No. 5925*.

[fsn31888-bib-0019] Ismail, A. , Gonçalves, B. L. , de Neeff, D. V. , Ponzilacqua, B. , Coppa, C. F. S. C. , Hintzsche, H. , … Oliveira, C. A. F. (2018). Aflatoxin in foodstuffs: Occurrence and recent advances in decontamination. Food Research International, 113, 74–85. 10.1016/j.foodres.2018.06.067 30195548

[fsn31888-bib-0020] Jager, A. , Tedesco, M. , Souto, P. , & Oliveira, C. (2013). Assessment of aflatoxin intake in São Paulo, Brazil. Food Control, 33(1), 87–92. 10.1016/j.foodcont.2013.02.016

[fsn31888-bib-0021] Joannis‐Cassan, C. , Tozlovanu, M. , Hadjeba‐Medjdoub, K. , Ballet, N. , & Pfohl‐Leszkowicz, A. (2011). Binding of zearalenone, aflatoxin B1, and ochratoxin A by yeast‐based products: A method for quantification of adsorption performance. Journal of Food Protection, 74(7), 1175–1185. 10.4315/0362-028X.JFP-11-023 21740721

[fsn31888-bib-0022] Karazhyan, R. , Shaker, S. I. , & Mehraban, S. M. (2017). Effect of Saccharomyces cerevisiae yeast on ruminal detoxification of aflatoxin B1. Journal of Veterinary Research, 72, 81–86.

[fsn31888-bib-0023] Karlovsky, P. , Suman, M. , Berthiller, F. , De Meester, J. , Eisenbrand, G. , Perrin, I. , … Dussort, P. (2016). Impact of food processing and detoxification treatments on mycotoxin contamination. Mycotoxin Research, 32(4), 179–205. 10.1007/s12550-016-0257-7 27554261 PMC5063913

[fsn31888-bib-0024] Ketney, O. , Santini, A. , & Oancea, S. (2017). Recent aflatoxin survey data in milk and milk products: A review. International Journal of Dairy Technology, 70(3), 320–331. 10.1111/1471-0307.12382

[fsn31888-bib-0025] Lv, J. , Huang, H. , Yu, L. U. , Whent, M. , Niu, Y. , Shi, H. , … Yu, L. L. (2012). Phenolic composition and nutraceutical properties of organic and conventional cinnamon and peppermint. Food Chemistry, 132(3), 1442–1450. 10.1016/j.foodchem.2011.11.135 29243634

[fsn31888-bib-0026] Milani, J. , & Heidari, S. (2017). Stability of ochratoxin A during bread making process. Journal of Food Safety, 37(1), e12283 10.1111/jfs.12283

[fsn31888-bib-0027] Milani, J. , & Maleki, G. (2014). Effects of processing on mycotoxin stability in cereals. Journal of the Science of Food and Agriculture, 94(12), 2372–2375. 10.1002/jsfa.6600 24497303

[fsn31888-bib-0028] Milani, J. , Nazari, S. , Saman, S. , Bamyar, E. , & Maleki, G. (2018). Effect of bread making process on aflatoxin level changes. Journal of Chemical Health Risks, 4(4).

[fsn31888-bib-0029] Mozaffary P. , Milani J. M. , & Heshmati (2019). The influence of yeast level and fermentation temperature on Ochratoxin A decrement during bread making. Food Science & Nutrition, 7(6), 2144–2150. 10.1002/fsn3.1059 31289662 PMC6593364

[fsn31888-bib-0030] Noroozi, R. , Sadeghi, E. , Yousefi, H. , Taheri, M. , Sarabi, P. , Dowati, A. , … Ghafouri‐Fard, S. (2019). Wound healing features of *Prosopis farcta*: In vitro evaluation of antibacterial, antioxidant, proliferative and angiogenic properties. Gene Reports, 17, 100482 10.1016/j.genrep.2019.100482

[fsn31888-bib-0031] Panda, P. , & Mehta, A. (2013). Aflatoxin detoxification potential of O cimum Tenuiflorum. Journal of Food Safety, 33(3), 265–272.

[fsn31888-bib-0032] Peng, C. , Wang, L. I. , An, F. , Zhang, L. E. , Wang, Y. , Li, S. , … Liu, H. (2015). Fate of ochratoxin A during wheat milling and some Chinese breakfast processing. Food Control, 57, 142–146. 10.1016/j.foodcont.2015.03.036

[fsn31888-bib-0033] Peng, Z. , Chen, L. , Zhu, Y. , Huang, Y. , Hu, X. , Wu, Q. , … Yang, W. (2018). Current major degradation methods for aflatoxins: A review. Trends in Food Science & Technology, 80, 155–166. 10.1016/j.tifs.2018.08.009

[fsn31888-bib-0034] Rad, A. H. , & Kasaie, Z. (2017). A comparative study on different methods for the evaluation of baker’s yeast bioactivity. International Journal of Food Properties, 20(1), 100–106. 10.1080/10942912.2016.1141297

[fsn31888-bib-0035] Rahimi, E. , Sadeghi, E. , Bohlouli, S. , & Karami, F. (2018). Fates of deoxynivalenol and deoxynivalenol‐3‐glucoside from wheat flour to Iranian traditional breads. Food Control, 91, 339–343. 10.1016/j.foodcont.2018.04.014

[fsn31888-bib-0036] Rastegar, H. , Shoeibi, S. , Yazdanpanah, H. , Amirahmadi, M. , Khaneghah, A. M. , Campagnollo, F. B. , & Sant’Ana, A. S. (2017). Removal of aflatoxin B1 by roasting with lemon juice and/or citric acid in contaminated pistachio nuts. Food Control, 71, 279–284. 10.1016/j.foodcont.2016.06.045

[fsn31888-bib-0037] Rushing, B. R. , & Selim, M. I. (2018). Aflatoxin B1: A review on metabolism, toxicity, occurrence in food, occupational exposure, and detoxification methods. Food and Chemical Toxicology.***** 10.1016/j.fct.2018.11.047 30468841

[fsn31888-bib-0038] Sadeghi, E. , Mesgarof, H. , Sharafi, K. , Almasi, A. , Oskoi, S. B. , & Meskini, H. (2013). Study of microbiological quality of flour produced in Kermanshah and Ilam factories (2010–2011). Iran Occupational Health, 10(5), 92–98.

[fsn31888-bib-0039] Schaarschmidt, S. , & Fauhl‐Hassek, C. (2018). The fate of mycotoxins during the processing of wheat for human consumption. Comprehensive Reviews in Food Science and Food Safety, 17(3), 556–593. 10.1111/1541-4337.12338 33350125

[fsn31888-bib-0040] Shetty, P. H. , Hald, B. , & Jespersen, L. (2007). Surface binding of aflatoxin B1 by *Saccharomyces cerevisiae* strains with potential decontaminating abilities in indigenous fermented foods. International Journal of Food Microbiology, 113(1), 41–46. 10.1016/j.ijfoodmicro.2006.07.013 16996157

[fsn31888-bib-0042] Uma Reddy, M. , Gulla, S. , & Nagalakshmi, A. (2010). Effect of fermentation on aflatoxin reduction in selected food products. Food Science Technology Nutrition, 4(1), 93–99.

[fsn31888-bib-0043] Valle‐Algarra, F. M. , Mateo, E. M. , Medina, A. , Mateo, F. , Gimeno‐Adelantado, J. V. , & Jiménez, M. (2009). Changes in ochratoxin A and type B trichothecenes contained in wheat flour during dough fermentation and bread‐baking. Food Additives and Contaminants, 26(6), 896–906. 10.1080/02652030902788938 19680965

[fsn31888-bib-0044] Velazhahan, R. , Vijayanandraj, S. , Vijayasamundeeswari, A. , Paranidharan, V. , Samiyappan, R. , Iwamoto, T. , … Muthukrishnan, S. (2010). Detoxification of aflatoxins by seed extracts of the medicinal plant, Trachyspermum ammi (L.) Sprague ex Turrill–structural analysis and biological toxicity of degradation product of aflatoxin G1. Food Control, 21(5), 719–725.

[fsn31888-bib-0045] Vidal, A. , Sanchis, V. , Ramos, A. J. , & Marin, S. (2016). The fate of deoxynivalenol through wheat processing to food products. Current Opinion in Food Science, 11, 34–39. 10.1016/j.cofs.2016.09.001

[fsn31888-bib-0046] Vijayanandraj, S. , Brinda, R. , Kannan, K. , Adhithya, R. , Vinothini, S. , Senthil, K. , … Velazhahan, R. (2014). Detoxification of aflatoxin B1 by an aqueous extract from leaves of Adhatoda vasica Nees. Microbiological Research, 169(4), 294–300. 10.1016/j.micres.2013.07.008 23928380

